# Role of Autophagy in Glycogen Breakdown and Its Relevance to Chloroquine Myopathy

**DOI:** 10.1371/journal.pbio.1001708

**Published:** 2013-11-12

**Authors:** Jonathan Zirin, Joppe Nieuwenhuis, Norbert Perrimon

**Affiliations:** 1 Department of Genetics, Harvard Medical School, Boston, Massachusetts, United States of America; 2 Howard Hughes Medical Institute, Harvard Medical School, Boston, Massachusetts, United States of America; University of Oslo, Norway

## Abstract

Several myopathies are associated with defects in autophagic and lysosomal degradation of glycogen, but it remains unclear how glycogen is targeted to the lysosome and what significance this process has for muscle cells. We have established a *Drosophila melanogaster* model to study glycogen autophagy in skeletal muscles, using chloroquine (CQ) to simulate a vacuolar myopathy that is completely dependent on the core autophagy genes. We show that autophagy is required for the most efficient degradation of glycogen in response to starvation. Furthermore, we show that CQ-induced myopathy can be improved by reduction of either autophagy or glycogen synthesis, the latter possibly due to a direct role of Glycogen Synthase in regulating autophagy through its interaction with Atg8.

## Abstract 2

A novel *Drosophila* model system of chloroquine myopathy reveals how glycogen is targeted to the lysosome and what the significance of this process is for muscle cells.

## Author Summary

Lysosomes are organelles that work as a disposal system for the cell. It is known that lysosomes can degrade glycogen and that defects in this function trigger the accumulation of vesicles containing glycogen in animals that lead to vacuolar myopathies—diseases that result in muscle weakness. However, it remains unclear how and why glycogen is degraded through this system, and what significance it has for the pathology of such diseases. Here, we addressed these questions by establishing a fruitfly model system to study glycogen autophagy in skeletal muscles. By feeding the flies chloroquine (CQ), we induce a vacuolar myopathy associated with massive accumulation of glycogen-filled vesicles, and assay the role of autophagy and glycogen metabolic enzymes in this process. We show that CQ-induced glycogen autophagy is completely dependent on the core conserved autophagy genes and that this autophagy is triggered by nutrient deprivation in a Tor-dependent manner. Interestingly, while glycogen autophagy and enzymatic glycogen breakdown can compensate for each other, concurrent inhibition of both systems blocks glycogen breakdown. Finally, we show that CQ-induced myopathy can be improved by reduction of either autophagy or glycogen synthesis, the latter possibly due to a direct role of glycogen synthase—the main enzyme involved in converting glucose to glycogen—in regulating autophagy through its interaction with the autophagosome.

## Introduction

Autophagy describes the sequestration of a cell's own cytoplasm and organelles into a closed double-membrane bound vesicle [1]. The completed vesicle, called the autophagosome, fuses with the lysosome, where its inner membrane and contents are degraded by hydrolases. The resulting degradation products are transported back to the cytoplasm where they can be reused for protein synthesis and ATP production. A major role of autophagy is therefore to liberate amino acids, fatty acids, and glucose that can be used to maintain cellular functions during stress and starvation. In mice, autophagy increases in most organs under starvation conditions, with muscles showing a particularly clear response [2]. Interestingly, glycogen-rich fast-twitch fibers induce autophagy much more robustly than oxidative slow-twitch fibers, suggesting a link between glucose metabolism and autophagy regulation.

Several myopathies are associated with accumulation of autophagic and lysosomal vesicles containing glycogen, but for most of them it remains unclear how glycogen metabolism connects to the pathology of the diseases [3],[4]. Among these are the hereditary primary lysosomal myopathies Pompe disease and Danon disease, infantile autophagic vacuolar myopathy, and the drug-induced vacuolar myopathies caused by treatment with chloroquine (CQ) or hydroxychloroquine [4]. The best characterized of these is the lysosomal storage disorder, Pompe disease, also known as glycogen storage disease type II. Pompe disease is caused by a mutation in the gene encoding acid a-glucosidase (GAA), an enzyme that localizes to the lysosome, and hydrolyzes glycogen to glucose [5]–[7]. Deficiencies of GAA in both humans and in mouse models lead to accumulation of lysosomes swollen with undegraded glycogen, as well as a secondary defect in the fusion between autophagosomes and lysosomes [8]–[10]. The resulting accumulation of autophagosomes and functional block of autophagy damages the muscle tissue and interferes with the efficacy of enzyme replacement therapy [11],[12]. The list of disorders classified as autophagic vacuolar myopaties (AVMs) is growing, although none but Danon and Pompe disease have been mapped to a causative gene [13].

More common than the myopathies described above, drug-induced myopathy may occur in as many as 12% of patients receiving antimalarial treatment with CQ [14]. CQ and its closely related analog hydroxychloroquine are 4-aminoquinoline compounds widely used to treat malaria, rheumatoid arthritis, and lupus erythematosus [15]–[17]. The drugs are highly lysosomotropic, causing an increase in lysosomal pH and inhibiting the fusion between autophagosomes and lysosomes [18],[19]. Thus, much like Pompe and Danon diseases, CQ myopathy may result from a blockage of autophagic flux indirectly caused by a lysosomal defect. Glycogen is a major component of the vacuoles in CQ myopathy patient biopsies, and a massive accumulation of glycogen filled autophagosomes was reported in denervated muscles of CQ-treated rats [20]–[22].

In addition to the glycogen-filled autophagosomes and lysosomes that appear during myopathies, mouse and rat neonates exhibit a dramatic autophagic sequestration of glycogen granules in the liver as well as in skeletal and cardiac muscles [23]–[25]. Lysosomal degradation of the large stores of glycogen in fetal tissues may be important for the survival of the animal during the starvation that occurs in the first few hours of life [26]. However, it remains unclear how the lysosomal degradation of glycogen during this period is regulated and whether it is substantially different from the lysosomal degradation of glycogen observed in skeletal muscle myopathy. Indeed, the mechanism of transport of glycogen to the lysosome is poorly understood in both cases.

The process of lysosomal glycogen degradation is sometimes referred to as glycogen autophagy (or glycophagy). Studies in yeast have identified 35 ATG (autophagy-related) genes, many of which are conserved in higher organisms [27],[28]. In all eukaryotes, autophagy is induced via the autophagy-related gene 1 (Atg1) complex. Autophagosomal membrane nucleation involves a complex containing Vps34 (the class III PI3K). Expansion of the autophagosome membrane requires two distinct sets of ubiquitin-like protein conjugation systems, Atg8 and Atg5–Atg12. Fusion with the lysosome requires the endocytic Rab proteins, HOPS complex, SNARE machinery, and the LAMP-1/LAMP-2 lysosomal membrane proteins [29]. The only *in vivo* genetic analysis of the role of these systems during glycogen transport to the lysosome was performed in the mouse model of Pompe disease, where glycogen accumulation in the lysosome was diminished in Atg7-deficient GAA KO muscles [12],[30],[31]. The consequence of genetic suppression of autophagy in muscles has not been reported for the other vacuolar myopathies and CQ myopathy, nor has the prevalence of glycogen autophagy been examined in neonatal autophagy gene mutants. Thus, it is not known to what extent the classic autophagic pathway is involved in glycogen autophagy, nor what effect autophagy suppression would have on the myopathy phenotypes.

A high level of conservation with higher organisms makes the *D. melanogaster* muscle an attractive system to study cellular processes, such as autophagy, that are involved in human disease [32],[33]. Here, we establish an *in vivo* model of glycogen autophagy in the *D. melanogaster* larval skeletal musculature, using CQ to simulate an autophagic myopathy that is completely dependent on the core autophagy genes. In this system, glycogen autophagy is triggered by nutrient deprivation, and is required for maximum rates of glycogen degradation in the muscle. Knockdown of *D. melanogaster* Glycogen synthase (GlyS), which is highly expressed in muscle, effectively blocks the formation of enlarged CQ-induced autophagosomes. This may be due to a direct function of GlyS, which localizes to autophagosomes and is able to form a complex with Atg8 in response to starvation. Formation of this complex is inhibited by mutations of either the GlyS putative LC3-interacting region (LIR) or an arginine predicted to be involved in glucose-6-phosphate binding.

## Results

### Effects of Chloroquine in *D. melanogaster* Larval Muscles

**Figure 1:**
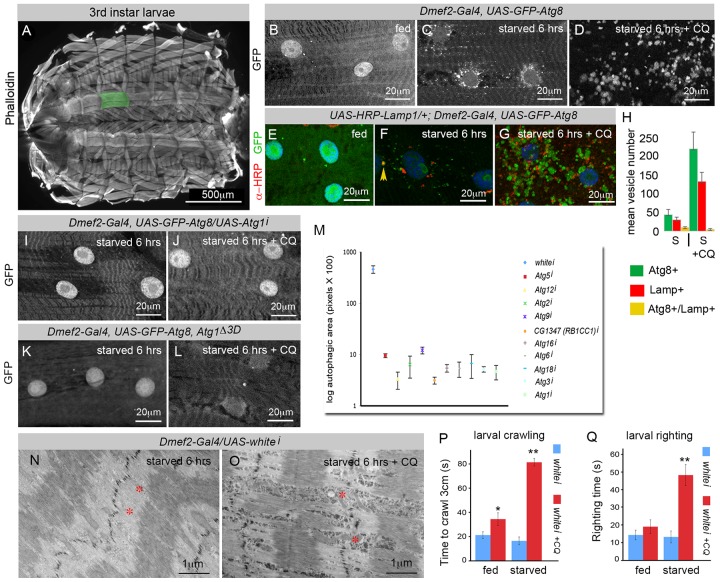
Chloroquine (CQ) treatment blocks autophagosome–lysosome fusion and induces myopathy in the larva. (A) Third instar larval skeletal musculature stained with Phalloidin (F-actin). In this and subsequent figures, we assayed the ventral longitudinal muscles (highlighted in green). (B–D) GFP–Atg8, overexpressed using the Dmef2–Gal4 driver, labels autophagosomes. *Dmef2–Gal4*, *UAS–GFP–Atg8* animals were fed on high-nutrient food (B), starved on low-nutrient food for 6 h (C), or starved on low-nutrient food +2.5 mg/ml CQ for 6 h (D). GFP–Atg8-labeled vesicles appeared only in the starved animals (C–D), localizing around the nucleus and between myofibers. (D) CQ treatment caused accumulation of bloated GFP–Atg8-labeled vesicles. (E–G) *Dmef2*–*Gal4*, *UAS*–*GFP*–*Atg8/UAS*–*HRP*–*Lamp1* animals were assayed for Lamp1 and Atg8 localization (anti-HRP, red; GFP, green; DAPI, blue). . (E) High-nutrient food suppressed formation of both GFP–Atg8 and HRP–Lamp1-labeled vesicles. (F) Colocalization of GFP–Atg8 and HRP–Lamp in animals starved on low-nutrient food. The yellow arrowhead points to a vesicle positive for both Atg8 and Lamp. (G) Addition of CQ to the starvation diet resulted in accumulation of both GFP–Atg8 and HRP–Lamp-labeled vesicles, but they failed to colocalize. (H) Quantification of the number of GFP–Atg8, HRP–Lamp1, or GFP–Atg8+HRP–Lamp1 vesicles in starved or starved +CQ muscles. (I–M) The core *Atg* genes are required for starvation-induced autophagy in both wild-type and CQ-treated skeletal muscles. *Dmef2*–*Gal4*, *UAS*–*GFP*–*Atg8/UAS*–*Atg1* larvae were starved on low-nutrient food for 6 h (I) or starved on low-nutrient food +2.5 mg/ml CQ for 6 h (J). Note that *Atg1* knockdown completely abolished the formation of GFP–Atg8-labeled autophagosomes (compare I–J to C–D). (K–L) *Dmef2*–*Gal4*, *UAS*–*GFP*–*Atg8*, *Atg1^Δ3d^* larvae failed to form GFP–Atg8 vesicles when starved or starved and treated with CQ. (M) Quantification of autophagy changes due to *Atg* gene knockdown in *Dmef2*–*Gal4*, *UAS*–*GFP*–*Atg8* larvae starved on low-nutrient food +2.5 mg/ml CQ for 6 h. Each of the 10 *UAS*–*Atg* RNAi transgenes tested caused a highly significant decrease (*p*<.01) in the total area of GFP–Atg8 vesicles. SEM is indicated, with *n* = 5 ventral longitudinal muscles from individual animals. (N–O) EM of muscles from *Dmef2*–*Gal4*, *UAS*–*whitei* larvae. Animals starved on low-nutrient food +2.5 mg/ml CQ (O) accumulated vesicles in the intermyofibril spaces (red asterisk), disrupting the integrity of the sarcomere compared to non-CQ-treated control muscles (N). (P) CQ treatment increased the larval crawling time of *Dmef2*–*Gal4*, *UAS*–*whitei* larvae in starved animals, and weakly in fed animals. (Q) CQ treatment increased the larval righting time of *Dmef2*–*Gal4*, *UAS*–*whitei* larvae in starved but not fed animals. For both locomotor assays, SEM is indicated for *n* = 10 larvae (*p<.05, **p<.01).

Our first goal was to establish an *in vivo* system to analyze the effects of CQ treatment on the larval skeletal musculature. Using a GFP-tagged version of the conserved lipid-conjugated ubiquitin-like protein Atg8, which localizes to autophagosomes in yeast, flies, and mammals [34], we assayed its quality as a marker of autophagy in dissected larval muscles. GFP–Atg8 expressed with *Dmef2–Gal4* had no effect on animal viability or on gross muscle morphology (unpublished data), and larvae fed on a standard diet (see Text S1) showed very few GFP–Atg8-labeled vesicle structures (Figure 1B). In contrast, in larvae starved on low nutrient food for 6 h, GFP–Atg8 localized to small punctae surrounding the nuclei and between the myofibrils (Figure 1C), consistent with observations in mice that autophagy increases in most organs under starvation conditions, with muscles showing a particularly clear response [2].

Prolonged treatment with CQ in mammals is associated with the onset of vacuolar myopathy, likely due to a defective autophagy–lysosome system. We treated third instar larvae with the drug to better visualize autophagic flux, and to more closely model the defects observed in AVMs such as CQ myopathy and Pompe and Danon diseases. In larvae treated with CQ and starved on low-nutrient food for 6 h, GFP–Atg8-labeled vesicles were much larger and more numerous than those in the nontreated muscle, but were similarly distributed around the nucleus and between myofibrils (Figure 1D).

One of the effects of CQ treatment in mammalian cells, and also of Pompe disease, is a defect in the fusion between autophagosomes and lysosomes. This causes a functional defect in autophagy and an accumulation of autophagosomes and lysosomes. To determine whether this was the case in larvae treated with CQ, we coexpressed GFP–Atg8 and the lysosomal membrane marker, HRP–Lamp1 (Figure 1E–H). In well-fed larvae, neither GFP–Atg8 nor HRP–Lamp1 localized to vesicles (Figure 1E). Starvation on low-nutrient food for 6 h induced the formation of small vesicles, some of which were labeled with both GFP–Atg8 and HRP–Lamp1, indicating that these are likely autolysosomes (Figure 1F,H). However, in larvae treated with CQ and starved, despite the accumulation of large and numerous GFP-labeled vesicles, we detected few vesicles that were co-labeled with HRP–Lamp1, indicating that CQ treatment effectively blocked fusion of autophagosomes and lysosomes (Figure 1G–H).

Next, we tested whether the formation of the GFP–Atg8 punctae observed in both untreated and CQ-treated larvae were dependent on a functional autophagy pathway. This is especially important given that Atg8 tends to be incorporated into intracellular protein aggregates, independent of autophagy. The association with aggregates includes endogenous Atg8 as well as ectopically expressed Atg8–GFP fusion protein. Thus, an Atg8 or Atg8–GFP positive punctae can represent either an aggregate or a bone fide autophagosome. Consistent with the latter interpretation, knockdown of *Atg1* was able to completely suppress the formation of GFP–Atg8 punctae in the muscles of both untreated (Figure 1I) and CQ-treated (Figure 1J) larvae starved on low-nutrient food for 6 h. Knockdown efficiency of the *Atg1* RNAi was confirmed by RT-PCR (Figure S3). Similarly, animals bearing a null allele of Atg1 were likewise unable to form GFP–Atg8 punctae (Figure 1K–L). We quantitated the suppression effect in the CQ-treated animals by measuring the total autophagic area per muscle cell, and tested 10 of the conserved core autophagy pathway genes, which all significantly inhibited the formation of GFP–Atg8 punctae (Figure 1M, Figure S1).

The accumulation of vesicles in muscles of CQ-treated larvae was strikingly similar to the phenotype observed in mammalian AVMs. We therefore set out to determine whether the larvae exhibited symptoms of myopathy. Electron microscopy (EM) on sectioned muscles from third instar starved larvae revealed that treatment with CQ did indeed cause disruption of the sarcomere structure compared with an untreated control (Figure 1N–O). This appears to occur through displacement of the sarcomere by enlarged vesicles in the intermyofibril spaces, similar to what has been reported for cases of CQ myopathy and Pompe disease in humans [35],[36]. To determine whether CQ treatment disrupted muscle function, we performed two tests of larval locomotion, the larval crawling assay (Figure 1P) and the larval righting assay (Figure 1Q). In both tests, CQ-treated larvae performed significantly worse than wild-type controls, but only in starved animals, suggesting that the accumulation of vesicles upon starvation may be responsible for a decline in muscle function.

### Autophagic Vesicles in Larval Muscles Are Loaded with Glycogen

**Figure 2:**
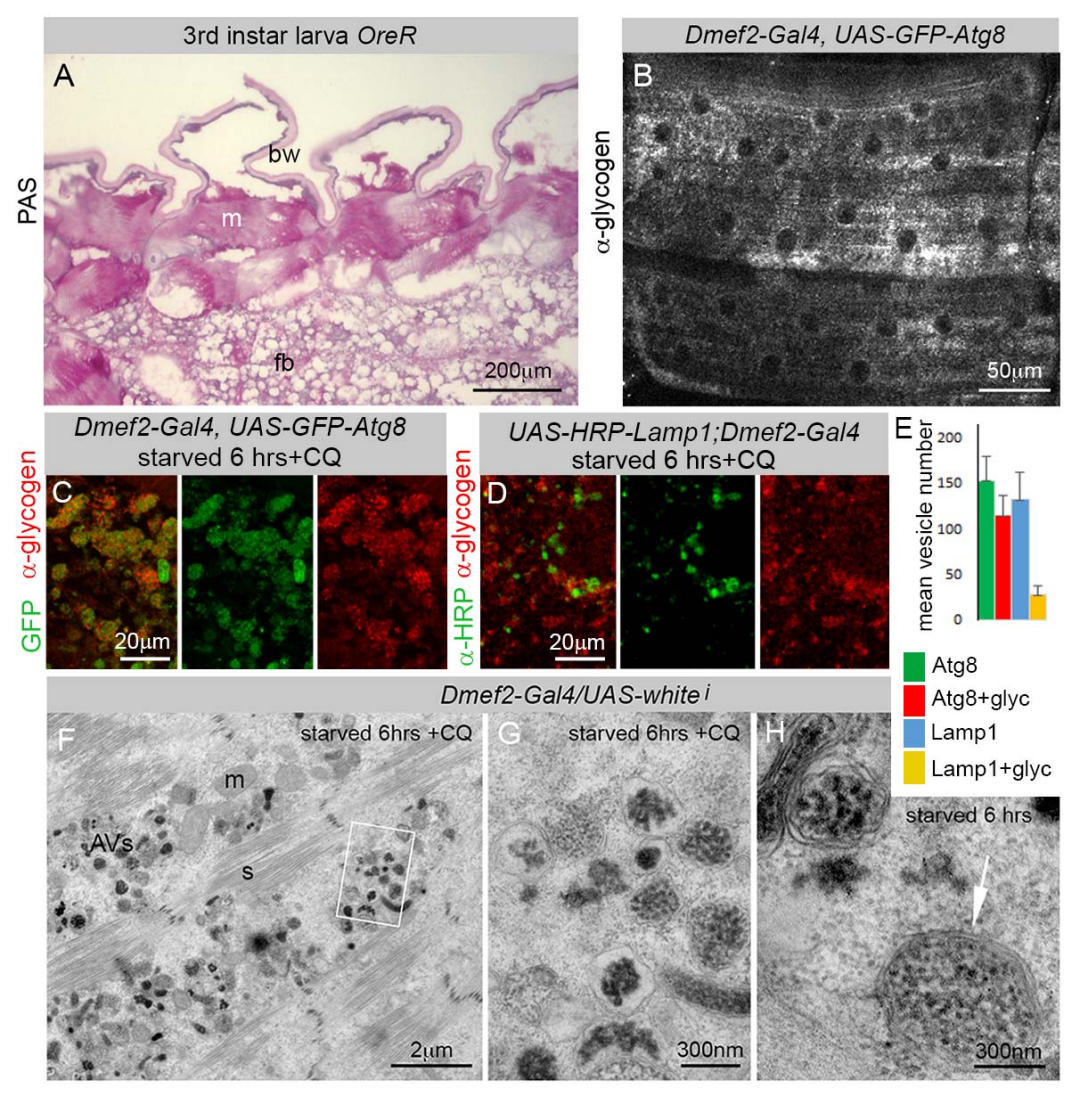
Autophagosomes in the larval muscle are filled with glycogen. (A) Sectioned third instar *OreR* larva stained with Periodic acid-Schiff (PAS). The muscles, but not the fat body, are stained purple, indicating high levels of glycogen (m, muscle; bw, body wall; fb, fat body). (B) Glycogen was also detected in muscle from *Dmef2*–*Gal4*, *UAS*–*GFP*–*Atg8* larvae, immunostained with an antiglycogen monoclonal antibody. (C) GFP–Atg8 vesicles colocalized with glycogen in *Dmef2*–*Gal4*, *UAS*–*GFP*–*Atg8* larvae starved on low-nutrient food +2.5 mg/ml CQ for 6 h (GFP, green; antiglycogen, red). (D) HRP–Lamp1 vesicles show less colocalization with glycogen in UAS–HRP–Lamp1;Dmef2–Gal4 larvae starved and treated with CQ. (E) Quantification of GFP–Atg8 or HRP–Lamp1 vesicles with glycogen. (F–G) EM from *Dmef2*–*Gal4*, *UAS*–*whitei* larvae starved on low-nutrient food +2.5 mg/ml CQ for 6 h. (F) Double- and single-membrane vesicles containing glycogen granules accumulated between myofibers (s, sarcomere; m, mitochondrion; AVs, autophagic vesicles). (G) Higher magnification view of region outlined in (E). (H) CQ treatment is not required for glycogen autophagy as seen in an EM from a *Dmef2*–*Gal4*, *UAS*–*whitei* larva starved on low-nutrient food for 6 h. Arrow points to double membrane.

In mammals glycogen is synthesized and stored in the muscle and liver. We analyzed glycogen storage in larvae using both the histological periodic acid-Schiff (PAS) stain (Figure 2A) as well as a monoclonal glycogen antibody (Figure 2B). Consistent with previous reports, we detected abundant glycogen stores in the third instar larval muscles, but not in the larval fat body, the tissue most closely analogous to the vertebrate liver and adipose tissue [37]. In addition, larvae treated with CQ and starved on low-nutrient food for 6 h showed a high degree of colocalization between GFP–Atg8 and glycogen (Figure 2C–E).

To ensure that the observed colocalization was indeed glycogen autophagy, we performed EM on sectioned muscles. Figure 2F–G demonstrates the massive build-up of vesicles in the starved and CQ-treated muscle. Consistent with results obtained by confocal microscopy, the vesicles accumulated near the nucleus and between the filaments of the myofibrils. Many of the vesicles were double-membraned, containing electron-dense glycogen granules. The same structures were also observed in non-CQ-treated muscles (Figure 2H), indicating that glycogen autophagy occurs normally in muscles, but with less frequency. Interestingly, although the larval skeletal muscle is filled with mitochondria (Figure 2F), we never observed any vesicles containing these organelles, nor did we ever observe colocalization between GFP–Atg8 and mitochondrial markers in the muscle (Figure S2). This is in contrast with several reports of autophagic vesicles containing mitochondria in other *D. melanogaster* tissues [38]–[40]. Thus, the larval muscle is the major site of glycogen storage in the larva, and muscle glycogen is the primary substrate of autophagic degradation.

### Glycogen Autophagy in the Larval Muscle Is Linked to Nutrient Levels and Is Blocked by CQ Treatment

**Figure 3:**
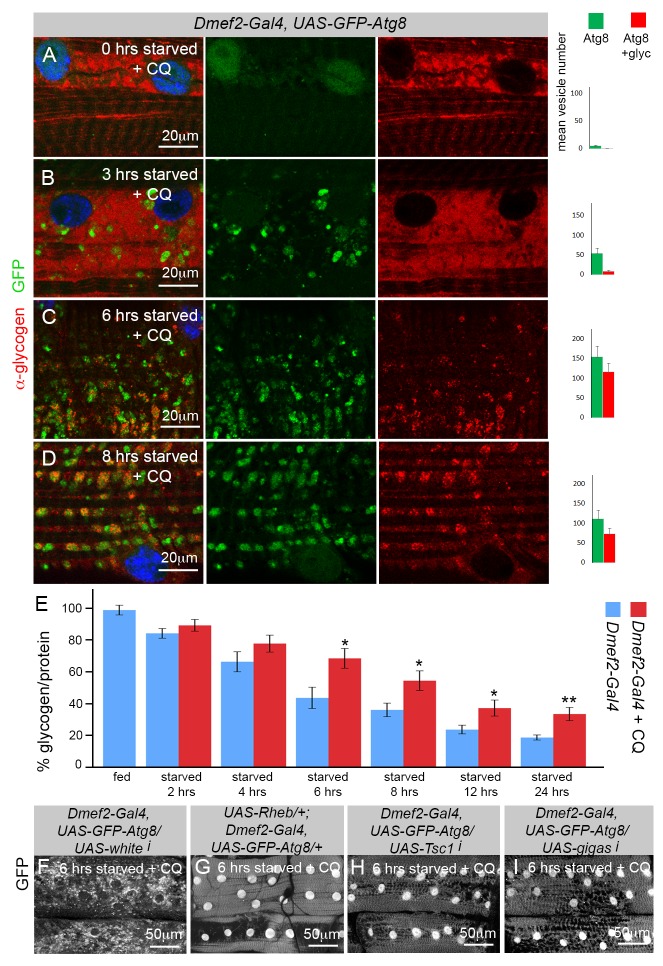
Degradation of glycogen via the autophagy lysosome system is regulated by nutrients and the Tor pathway. (A–D) Time course of autophagy induction in *Dmef2*–*Gal4*, *UAS*–*GFP*–*Atg8* muscles, accompanied by quantification of GFP–Atg8 and glycogen colocalization. Animals were fed for 18 h in high-nutrient food +2.5 mg/ml CQ, then starved on low-nutrient food +2.5 mg/ml CQ for 0–8 h (antiglycogen, red; GFP, green; DAPI, blue). (A) At time point 0, following 18 h in high-nutrient food +CQ, the muscles contained large amounts of glycogen with no apparent autophagy. (B) At 3 h of starvation, glycogen stores were still high, and GFP–Atg8-labeled vesicles began to appear. (C–D) At 6 and 8 h of starvation, the majority of GFP–Atg8-labeled vesicles colocalized with glycogen. (E) Time course of glycogen levels in *Dmef2*–*Gal4* carcasses (muscle+body wall). Animals were fed for 24 h in high-nutrient food, then starved on low-nutrient food +/− 2.5 mg/ml CQ for 0–24 h. Starvation caused reduction of glycogen levels in both untreated and CQ-treated larvae over time. However, after 6 h of starvation, CQ treatment significantly increased glycogen levels compared to controls. SEM is indicated for *n* = 5–8 samples (*p<.05, **p<.01). (F–G) activation of the Tor pathway blocked autophagy in the muscles from larvae starved on low-nutrient food +2.5 mg/ml CQ for 6 h. (F) Autophagy levels were high in control *Dmef2*–*Gal4/UAS*–*whitei* larvae. Muscles from (G) *UAS-Rheb/+; Dmef2-Gal4/+*, (H) *Dmef2*–*Gal4/UAS*–*Tsc1i*, and (I) *Dmef2*–*Gal4/UAS*–*gigi* all failed to induce autophagy.

Although the degradation of glycogen by the lysosome was discovered in the 1960s, little is known about its regulation [7],[24]. In particular, it is not clear whether the induction of autophagy in the muscle and the localization of glycogen in the autophagosomes are subject to regulation by nutrient availability and the Tor pathway. Thus, we performed a time course of glycogen autophagy at 0–8 h of starvation in CQ-treated larvae (Figure 3A–D). Animals were first fed for 18 h on high-nutrient food +CQ, then transferred to low-nutrient starvation food +CQ, and then dissected and stained following each time point. At 0 h of starvation, there was abundant glycogen in the muscle but few GFP–Atg8 punctae, indicating that the rich diet was able to completely suppress autophagy even in the presence of CQ (Figure 3A). At 2–3 h of starvation, autophagosomes began to appear in the muscle, although glycogen remained detectable at a high level (Figure 3B). By 6–8 h much of the glycogen had been degraded, and what remained was now mostly localized to the GFP–Atg8 vesicles (Figure 3C–D). Altogether, these data indicate that muscle glycogen stores are depleted by starvation and that the induction of autophagy and the localization of glycogen within the autophagosomes are regulated by nutrient intake.

To more accurately quantitate these effects, we performed a starvation time course experiment on larvae with or without CQ treatment (Figure 3E). Following starvation we collected the carcasses, and measured the glycogen content by enzymatic assay (see Materials and Methods). Glycogen levels diminished over time in both untreated and CQ-treated larvae. However, the latter group showed a significantly reduced rate of glycogen loss, and heightened levels of glycogen persisted even after 24 h of starvation. This may represent glycogen that remains trapped, undegraded, in the autophagosomes and lysosomes of CQ-treated larvae.

The Tor kinase pathway links cellular nutritional status to metabolism, growth, and autophagy [41]. Tor activity is inhibited by the Tsc1/Tsc2 complex, which in turn inhibits the small G-protein Rheb, and GTP-bound Rheb binds to and activates Tor [42]. As TOR signaling represses the formation of autophagosomes by inhibition of Atg1, a function conserved from yeast to mammals [43], we tested whether the induction of autophagy in muscle is subject to TOR regulation by overexpressing *Rheb* with the *Dmef2–Gal4* driver. Strikingly, the localization of GFP–Atg8 to autophagosomes in starved/CQ-treated muscles was completely blocked by *Rheb* overexpression compared to controls (Figure 3F–G). Similar to *Rheb* overexpression, knockdown of *Tsc1* and *gigas (Tsc2)* dramatically inhibited autophagy (Figure 3H–I). Therefore, glycogen autophagy in the larval muscle is linked to nutrient levels via the TOR pathway.

### Glycogen Breakdown Requires Either Functioning Autophagy or Glycogenolysis Systems

**Figure 4:**
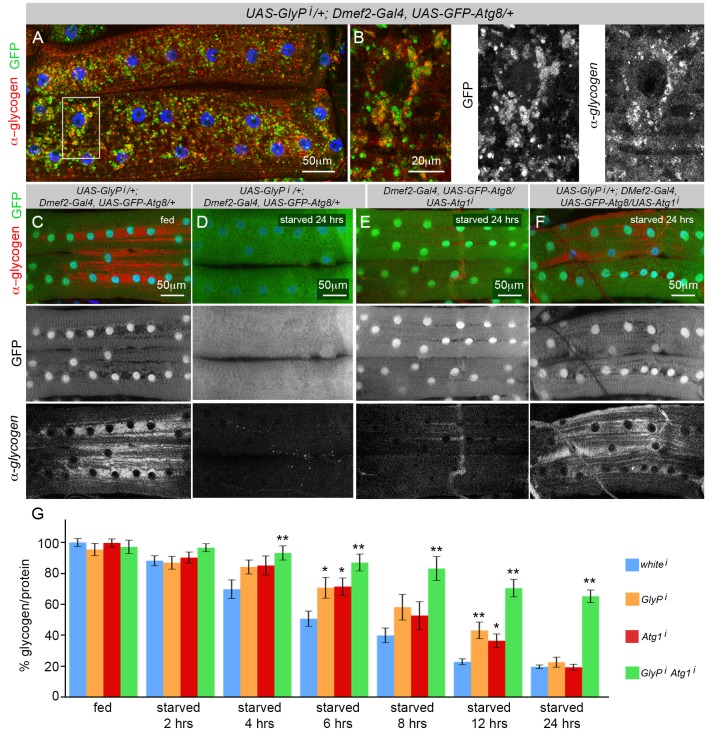
Autophagy and glycogenolysis compensate for each other, but both systems are required for maximal glycogen catabolism. (A–B) Glycogen phosphorylase is not required for glycogen autophagy (antiglycogen, red; GFP, green; DAPI, blue). (A) *UAS*–*GlyPi/+; Dmef2*–*Gal4*, *UAS*–*GFP*–*Atg8/+* larvae starved on low-nutrient food +2.5 mg/ml CQ for 6 h exhibited high levels of colocalization between GFP–Atg8 and glycogen. (B) Higher magnification of region outlined in (A). (C–F) *Dmef2*–*Gal4*, *UAS*–*GFP*–*Atg8* larvae with *GlyP* and/or *Atg1* knockdown were fed on high-nutrient food for 18 h before being starved on low-nutrient food (antiglycogen, red; GFP, green; DAPI, blue). (C) *UAS*–*GlyPi/+; Dmef2*–*Gal4*, *UAS*–*GFP*–*Atg8/+* larval muscle contained high levels of glycogen prior to starvation, indicating no defect in glycogen synthesis. (D) Following 24 h starvation *UAS*–*GlyPi/+; Dmef2*–*Gal4*, *UAS*–*GFP*–*Atg8/+* muscles contained no glycogen detectable by antibody staining. (E) Following 24 h of starvation *Dmef2*–*Gal4*, *UAS*–*GFP*–*Atg8/UAS*–*Atg1i* muscles contained no glycogen. (F) Double-mutant larvae *UAS*–*GlyPi/+; Dmef2*–*Gal4*, *UAS*–*GFP*–*Atg8/UAS*–*Atg1i* larval muscles contained high levels of glycogen after 24 h of starvation, indicating an inability to break down glycogen. (G) Time course of glycogen levels in *Dmef2*–*Gal4* carcasses (muscle+body wall) with expression of UAS–RNAi transgenes targeting *white*, *GlyP*, *Atg1*, or *GlyP+Atg1*. Simultaneous knockdown of *GlyP* and *Atg1*, but not either gene alone, significantly reduced glycogen degradation compared to the *white* control after 24 h of starvation, consistent with immunostaining (C–F). Between 6 and 12 h of starvation, individual knockdown of *GlyP* or *Atg1* caused a significant increase in glycogen levels, indicating a reduced rate of glycogen degradation. SEM is indicated for *n* = 5–8 samples. The *p* values were calculated relative to white RNAi control at each time point (*p<.05, **p<.01).

One of the critical unanswered questions related to glycogen and autophagy is how the lysosomal degradation of glycogen relates to the enzymatic degradation of glycogen via the action of glycogen phosphorylase. *D. melanogaster* has a single gene encoding glycogen phosphorylase, *GlyP*, which has a high degree of sequence homology to the mammalian enzymes. To test whether *GlyP* is required for glycogen autophagy, RNAi targeting *GlyP* was expressed along with *GFP–Atg8* using *Dmef2–Gal4*. This reduced the *GlyP* expression level in the muscle by more than 90% (Figure S3). Third instar larvae starved on low-nutrient food for 6 h and treated with CQ exhibited the same colocalization of GFP–Atg8 and glycogen (Figure 4A–B) as was observed in control *Dmef2–Gal4*, *UAS–GFP–Atg8* muscles (Figure 2C–D).

Next, we assayed the ability of muscles deficient in *GlyP* to break down glycogen. Prior to dissection, larvae were fed for 24 h on high-nutrient food. Muscles from *GlyP* knockdown larvae given this diet exhibited large deposits of glycogen throughout the skeletal muscle cells (Figure 4C). Following the rich food diet, larvae were transferred to low-nutrient starvation food for 24 h prior to dissection. Surprisingly, after the starvation period, glycogen was almost completely undetectable in muscles from the *GlyP* knockdown larvae (Figure 4D). Likewise, knockdown of *Atg1* had no effect on the ability of muscle cells to break down glycogen in this context (Figure 4E), suggesting that during starvation both glycogenolysis and autophagy are sufficient to break down glycogen such that neither is absolutely required. To test this hypothesis we knocked down both *GlyP* and *Atg1* simultaneously. Despite 24 hrs of starvation, these larvae maintained high levels of glycogen in their muscles, indicating that glycogen breakdown requires either a functioning autophagy or glycogenolysis system (Figure 4F).

In order to quantitate these effects, we performed a starvation time course experiment on larvae expressing RNAs targeting *white*, *Atg1*, *GlyP*, or *Atg1+GlyP* (Figure 4G). Consistent with the immunofluorescence results above, we found that after 24 h starvation, only *Atg1+GlyP* RNAi significantly inhibited glycogen degradation. However, at earlier time points (4–12 h starvation), individual targeting of *Atg1* or *GlyP* also reduced glycogen degradation, suggesting that each contributes to the maximal rate of degradation during this period.

### Glycogen Synthase Is Required for CQ-Induced Autophagosome Enlargement

**Figure 5:**
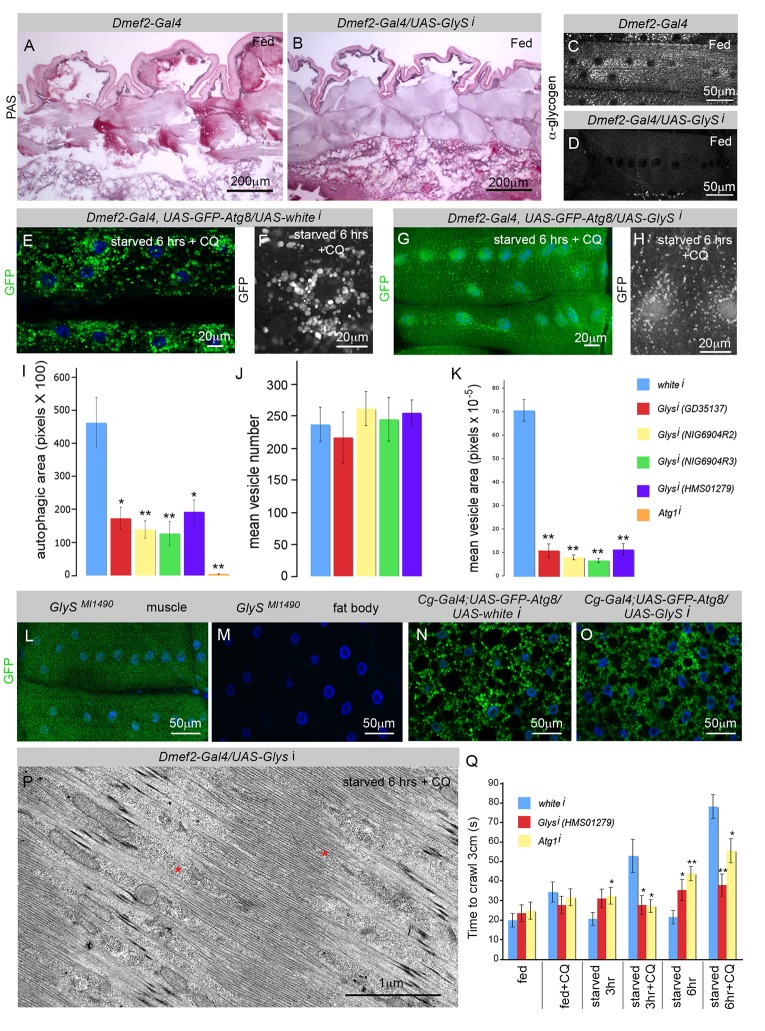
Glycogen synthase knockdown inhibits autophagosome growth and improves CQ-induced myopathy phenotype. (A–D) *Glycogen synthase* (*GlyS*) is required for glycogen synthesis in *D. melanogaster* muscles. PAS staining for glycogen was absent in *Dmef2*–*Gal4/UAS*–*GlySi* muscles (B) compared to control *Dmef2*–*Gal4/UAS*–*whitei* muscles (A). Antiglycogen immunostaining for glycogen was absent in *Dmef2*–*Gal4/UAS*–*GlySi* muscles (D) compared to *Dmef2*–*Gal4/UAS*–*whitei* control muscles (C). (E–K) *GlyS* is required for the formation of large CQ-induced autophagosomes. Vesicles are much smaller in *Dmef2*–*Gal4*, *UAS*–*GFP*–*Atg8/UAS*–*GlySi* larval muscle starved 6 h in low-nutrient food +2.5 mg/ml CQ (G) than in control *Dmef2*–*Gal4*, *UAS*–*GFP*–*Atg8/UAS*–*whitei* larval muscle (E). (F, H) The difference in autophagosome size is clearly evident at high magnification. (I–K) Quantification of autophagy changes due to *GlyS* gene knockdown in *Dmef2*–*Gal4*, *UAS*–*GFP*–*Atg8* larvae starved on low-nutrient food +2.5 mg/ml CQ for 6 h. SEM is indicated, with *n* = 5 (I) or *n* = 10 (J–K) ventral longitudinal muscles from individual animals (*p<.05, **p<.01). (I) Each of the four *UAS-GlyS* RNAi transgenes tested caused a significant decrease in the total area of GFP–Atg8 vesicles in the muscle compared to the *UAS*–*whitei* control. (J) Vesicle number was unchanged by *GlyS* knockdown. (K) *UAS*–*GlyS* RNAi caused a highly significant decrease in the mean vesicle size (area) compared to the control. (L–M) *GlyS* gene expression, monitored using a MiMIC transposon insertion (*MI01490*), showed expression in the larval muscle (L) but not the fat body (M) (green, GFP; blue, DAPI). (N–O) Larvae were starved on low-nutrient food for 6 h prior to dissection of the fat bodies. Autophagy in *Cg-Gal4/+; UAS*–*GFP*–*Atg8/UAS*–*GlySi* (O) was not substantially different from autophagy in *Cg-Gal4/+; UAS*–*GFP*–*Atg8/UAS*–*whitei* control fat bodies (GFP, green; DAPI, blue). (P) EM of muscle from *Dmef2*–*Gal4/UAS*–*GlySi* animal starved on low-nutrient food +CQ. Note that the intermyofibril spaces (red asterisk) and sarcomere structure are not distorted. (Q) *GlyS* or *Atg1* knockdown significantly improved the crawling time of larvae treated with CQ and starved for 6 h. SEM is indicated for *n* = 10 larvae. The *p* values were calculated relative to *white* RNAi control larvae (*p<.05, **p<.01).

The polymerization of glucose molecules into a glycogen chain is catalyzed by glycogen synthase, the rate-limiting enzyme of glycogenesis. *D. melanogaster* has a single glycogen synthase ortholog (*CG6904*), which we refer to as *GlyS*. Consistent with its proposed role in glycogen synthesis, muscles expressing RNAi targeting *GlyS* showed a dramatic decrease in PAS staining and antiglycogen immunostaining compared to controls (Figure 5A–D). We tested four *UAS*-*GlyS* RNAi constructs and each caused over 60% gene knockdown in the larval muscle when expressed by *Dmef2-Gal4* (Figure S3). To determine whether GlyS levels affected autophagosome formation in the muscle irrespective of whether the vesicles contain glycogen or not, we examined GFP–Atg8 localization in starved and CQ-treated control versus GlyS knockdown muscles. Strikingly, we observed dramatically reduced GFP–Atg8 vesicle localization in the latter (Figure 5E–H). To analyze this more closely, we quantitated the autophagic area, vesicle number, and vesicle size in CQ-treated control versus GlyS knockdown muscles, and found that almost all of the effects of GlyS knockdown are due to reduced vesicle size, not number. Thus GlyS is required for the formation of the bloated autophagic vesicles formed during CQ treatment.

Next, we tested whether the effect of *GlyS* on autophagy was limited to muscle cells or extended to the *D. melanogaster* liver analog, the fat body. Using PAS staining, we have shown that the muscle is the major site of glycogen storage in the larva (Figure 2A–B), however the fat body does contain some relatively low level of glycogen (Figure 2A–B, Figure 5A). We examined the expression of the *GlyS* gene using a transposon insertion (*MI01490*) that directs GFP expression under control of the endogenous *GlyS* regulatory elements. *MI01490* larvae had strong GFP expression in the third instar skeletal muscle (Figure 5L), but undetectable levels in the same stage fat body (Figure 5M), consistent with the much higher glycogen levels in the muscle. Knockdown of *GlyS* using the fat-body–specific Gal4 driver, *Cg*–*Gal4*, caused no appreciable effect on the accumulation of GFP–Atg8-labeled autophagosomes (Figure 5N–O) of larvae treated with CQ and starved on low-nutrient food for 6 h. Thus, the effect of GlyS on autophagy is tissue specific and not a general property of all cells.

Given the importance of GlyS to autophagosome formation, we wondered whether GlyS levels might ameliorate the myopathy observed in larvae treated with CQ (Figure 1N–O). EM of *Dmef2*–*Gal4/UAS*–*GlyS RNAi* larval skeletal muscle, starved and treated with CQ (Figure 5P), showed a much improved sarcomere structure compared to control larvae (Figure 1N), with much less distortion of the intermyofibrillar spaces. We also examined the effect of *GlyS* knockdown and *Atg1* knockdown on larval locomotion in the crawling assay (Figure 5Q). Larvae were fed, starved 3 h, or starved 6 h, with or without CQ. In fed larvae there was little difference between the *white*, *GlyS*, or *Atg1* knockdown, irrespective of CQ treatment. At 3 h starvation, both *GlyS* and *Atg1* knockdown significantly improved the crawling time of CQ-treated larvae. This remained true at 6 h of starvation, however the effect was less pronounced. Indeed, *Atg1* knockdown, and especially *GlyS* knockdown, began to negatively affect locomotor function at 6 h starvation even without CQ treatment. In the case of GlyS, this may be due to its essential function in making glycogen rather than a direct effect on autophagosome formation.

### Mutations in the LC3-Interacting Region (LIR) or Glucose-6-Phosphate (G-6-P) Binding Region of GlyS Prevent Its Interaction with Atg8

**Figure 6:**
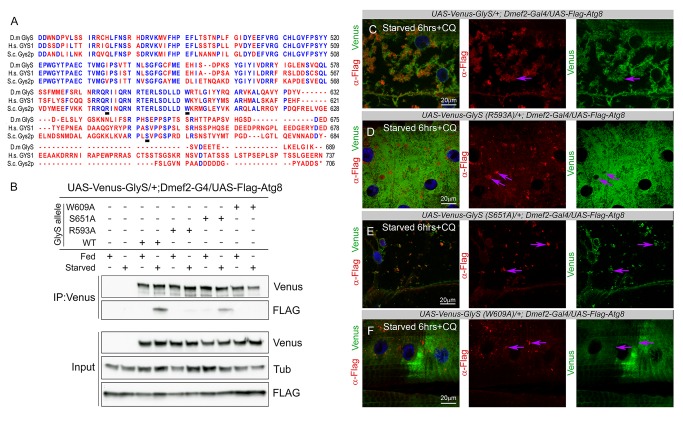
Interaction and colocalization of Glycogen synthase with Atg8 is disrupted in R593A and W609A mutants. (A) Protein sequence alignment of the C-terminal region of *D. melanogaster*, human, and yeast Glycogen synthases. Identical residues are blue; all other residues are red. Conserved in all three species, R593, W609, and S651 are underlined. (B) Western blot/Co-immunoprecipitation (co-IP) showing that Flag–Atg8 binds to a Venus–GlyS or Venus–GlyS (S651A) protein complex in response to starvation. Flag–Atg8 is unable to co-IP with either Venus–GlyS (R593A) or Venus–GlyS (W609A). Venus–GlyS and Venus–GlyS mutants were co-IP'd from muscle lysate from *Dmef2*–*Gal4/UAS*–*Flag*–*Atg8* or *UAS*–*Venus*–*GlyS(WT or mutant)/+;Dmef2*–*Gal4/UAS*–*Flag*–*Atg8* third instar larvae. These were fed on high-nutrient food for 18 h, and then transferred to fresh high-nutrient food or low-nutrient food for 6 h. (C–F) *UAS*–*Venus*–*GlyS (WT or mutant)/+;Dmef2*–*Gal4/UAS*–*Flag*–*Atg8* larvae were treated with starved 6 h in low-nutrient food +2.5 mg/ml CQ (Venus, green; α-Flag, red). Purple arrows mark examples of the presence or absence of colocalization. (C) Venus–GlyS was localized predominantly to the Flag-labeled autophagosomes, with weak staining in the rest of the cytoplasm. (D) Venus–GlyS (R593A) was found throughout the cytoplasm and did not colocalize with autophagosomes. (E) Venus–GlyS (S651A) was localized to the autophagosomes. (F) Venus–GlyS (W609A) did not colocalize with the Flag-labeled autophagosomes.

Interestingly, the human glycogen synthase muscle isoform was identified by mass spectroscopy as a potential interactor of GABARAPL1, one of the human orthologs of Atg8 [44], suggesting a possible mechanistic link between the autophagy machinery and glycogen. By binding ATG8 family members, some proteins can act as receptors that link their cargo to the nascent autophagosome membrane via an LC3-interacting region (LIR) with a consensus sequence of W/F-X-X-L/I/V, preceded by acidic residues. [45]. We identified three putative LIR motifs conserved between *D. melanogaster* GlyS and its mammalian orthologs: VAHFHE (residues 187–192), EFQNL (residues 303–307), and DWRTL (residues 608–612). We focused on the last of these as it contains both tryptophan and leucine, the canonical residues at their respective positions. To determine whether GlyS and Atg8 can interact in the larval muscle, we therefore generated a wild-type GlyS overexpression construct as well as overexpression constructs with a mutation at the critical tryptophan of the LIR (W609A). Additionally, to analyze whether the activation state of GlyS might be important for its role in autophagy, we generated additional constructs with mutations in the G-6-P binding region (R593A) and at the first GSK3B phosphorylation site (S651A) (Figure 6A) [46]. These UAS–Venus-tagged forms of *D. melanogaster GlyS* and *GlyS* mutants were expressed specifically in muscle with *Dmef2–Gal4/UAS–Flag–Atg8*. *UAS–Venus–GlyS (WT or mutants);Dmef2–Gal4/UAS–Flag–Atg8* larvae were fed on high-nutrient food or starved on low-nutrient food for 6 h and then lysed and immunoprecipitated with anti-GFP nanobodies. Flag–Atg8 did not Co-IP with Venus–GlyS in the fed animals, but starvation consistently caused the proteins to Co-IP (Figure 6B). The S651A mutant GlyS, which is predicted to be resistant to suppression by GSK3B phosphorylation [47],[48], was likewise able to interact with Flag–Atg8 under starvation conditions (Figure 6B). Neither the W609A mutant nor R593A mutant were able to Co-IP Flag–Atg8 in either nutritional state (Figure 6B).

We next overexpressed the tagged forms of GlyS in the larval muscle, and assayed for their localization with respect to Flag–Atg8 (Figure 6C–F). Consistent with the results of the Co-IP experiments, we observed colocalization of both Venus–GlyS (Figure 6C) and Venus–GlyS (S651A) (Figure 6E) with Flag–Atg8 in muscles from animals starved and treated with CQ. In contrast, Venus–GlyS (R593A) (Figure 6D) and Venus–GlyS (W609A) (Figure 6F) were found throughout the cytoplasm and did not colocalize with autophagosomes in muscles from starved and CQ-treated animals. Taken together these results indicate that GlyS and Atg8 can interact in *D. melanogaster* muscles, and that this interaction is regulated by nutritional status. Furthermore, the failure of the W609A mutant to interact or colocalize with Atg8 raises the possibility that the GlyS–Atg8 interaction might occur directly via the GlyS LIR.

## Discussion

Since the discovery in 1963 that Pompe disease corresponds to a deficiency in the lysosomal enzyme acid a-glucosidase, followed by the identification of several more lysosomal storage disorders and vacuolar myopathies, defects in glycogen metabolism have been linked to myopathies [3],[4],[7]. Strikingly, almost 50 years later, it remains unclear how glycogen metabolism and the autophagy–lysosome system interact in muscle cells. Here, we have established a *D. melanogaster* model to study glycogen autophagy in skeletal muscles, using chloroquine (CQ) to simulate an autophagic myopathy that is completely dependent on the core autophagy genes. We discovered that glycogen was sequestered into autophagic vesicles in response to starvation, and that this could be suppressed via the Tor pathway. We found that glycogen autophagy was able to compensate for a deficiency of glycogen phosphorylase over the course of 24 h of starvation, but that both degradation systems were required for maximum efficiency of glycogen breakdown during the first 12 h of starvation. Using our CQ-induced myopathy model system, we showed that reduction of either autophagy or glycogen synthesis dramatically improved the locomotor function of the drug-treated animals. Finally, we examined the relationship between GlyS and Atg8, and discovered that the two proteins can interact in the muscle cell and that this interaction depends on conserved arginine and tryptophan residues in the glucose-6-phosphate binding region and putative LIR of GlyS.

### Chloroquine-Induced Myopathy: A Model to Study Glycogen Autophagy in Larval Muscles

Using the muscle-specific Dmef2–Gal4 driver and GFP–Atg8 to label autophagosomes, we found that simply adding CQ to the food induced a dramatic increase in the size of autophagosomes, causing a dramatic distortion of the sarcomere as enlarged autophagosomes filled the intermyofibrillar spaces, causing them to bulge (Figure 1). This phenotype is strikingly similar to published reports of CQ-induced myopathy and Pompe disease in humans [35],[36]. Second, we assayed the locomotor function of treated larvae and found that CQ dramatically reduced the animals' crawling ability (Figure 1O–P). We cannot rule out that the effects of the drug on the nervous system could have played a role in this phenotype. However, the fact that we were later able to suppress the locomotor defects through muscle-specific genetic knockdowns indicates that at least some of this phenotype was due to the myopathy.

Using our CQ-induced myopathy model we set out to carefully examine the substrate of the accumulated autophagosomes. We thus identified glycogen as a major substrate of autophagy in the larval muscle by immunofluorescence and electron microscopy in both CQ-treated and untreated larvae (Figure 2). To further analyze the nature of the cargo during CQ-induced autophagy, we tested several other potential substrates for their presence in CQ-induced autophagosomes (Figure S2). We never detected the presence of mitochondria in the autophagosomes, indicating that autophagy was, to some extent, selective, as the cytoplasm of the larval muscle is rich in mitochondria. Further, we never observed autophagosomes containing the sarcomeric protein filamin, one of the previously reported substrates of autophagy in *D. melanogaster* adult flight muscle [49]. The flight muscle, a highly oxidative type of muscle, has also been used to model the function of the autophagy pathway during aging, where it seems to target ubiquitylated protein aggregates for degradation [50]. We found that a small number of autophagosomes stained positive for polyubiquitin in the larval muscle, but much fewer than those containing glycogen. Ubiquitin labeling is therefore unlikely to play a major role in the targeting of glycogen to the autophagosome. Taken together, these observations highlight the fundamental difference between autophagy in the highly glycolytic larval muscle and the oxidative flight muscle. This phenomenon extends to mammals, where large accumulations of autophagosomes are seen in glycolytic type II muscle fibers, but not in oxidative type I fibers, in the mouse model of Pompe disease [51]. Our data raise the possibility that differences in the autophagic substrate might underlie this phenotype.

The only previous analyses of the *Atg* genes and glycogen autophagy was in the mouse Pompe disease model, and was limited to mutations in two genes, *Atg7* and *Atg5*, which surprisingly had different effects on the amount of lysosmal glycogen [12],[30],[31]. Using transgenic RNAi lines, we were able to target components from each of the major Atg protein complexes (Figure 1I–K) [27],[28],[32]. Atg1, the *D. melanogaster* ortholog of mammalian Ulk1/2 kinase, and CG1347/Atg17, the ortholog of the Ulk-interacting protein RB1CC1/FIP200, function together and form a complex that is essential for autophagosome formation. The Atg12 complex (Atg5, Atg12, Atg16) localizes to the phagophore and is important for vesicle elongation. The E2-like enzyme, Atg3, is required for the conjugation of Atg8 to phosphatidylethanolamine (PE). Atg6 is required for the induction of autophagy as part of the class III phosphatidylinositol 3-kinase complex. Cycling of Atg9 between the PAS and peripheral sites is essential for autophagosome formation, and depends on the Atg9 interacting proteins, Atg2 and Atg18. Knockdown of any of these genes completely blocked autophagy in the *D. melanogaster* muscle, even in larvae starved and treated with CQ, indicating that the core conserved autophagy machinery is absolutely required for glycogen autophagy.

### The Function of Autophagic Glycogen Degradation in Muscles

One of the critical unanswered questions related to glycogen and autophagy is how the lysosomal degradation of glycogen relates to the enzymatic degradation of glycogen via glycogenolysis. In the latter, glycogen phosphorylase catalyzes the rate-limiting cleavage of glucose monomers from the end of a glycogen branch. Mutations in the muscle isoform of mammalian glycogen phosphorylase (PYGM) cause glycogen storage disease type V (also known as McArdle's Disease), while mutations in the liver isoform (PYGL) cause glycogen storage disease type VI (also known as Hers' disease) [52],[53]. By knocking down the *D. melanogaster* glycogen phosphorylase gene, *GlyP*, and *Atg1*, we showed that neither glycogenolysis nor autophagy were required for glycogen breakdown over the course of 24 h of starvation (Figure 4). Muscles deficient in both systems, however, were unable to degrade glycogen, indicating that glycogenolysis and autophagy are the only two routes of glycogen degradation available to the muscles, and that transport of glycogen to the lysosome for degradation requires a functioning autophagy system.

When we more closely examined the effects of individually knocking down *Atg1* or *GlyP*, we found that during the first 12 h of starvation the mutant muscles maintained higher levels of glycogen than wild-type controls. A similar effect was also observed in CQ-treated muscles, which have a functional block in autophagy (Figure 3). CQ treatment consistently caused an increase in glycogen levels over controls (Figure 3E). This effect was observed even after 24 h of starvation, which we suspect is due to the persistence of large numbers of autophagosomes filled with glycogen, protected from both glycogenolysis and the lysosome. We conclude that although autophagy and enzymatic glycogen breakdown can compensate for each other over the long term, in the first 12 h after starvation both systems are required for the maximum efficiency of glycogen breakdown.

Although these results suggest that glycogen autophagy in the *D. melanogaster* muscle targets the same stores of glycogen as GlyP in response to starvation, it is possible that in some cases, autophagy specifically targets mis-branched glycogen. Lending support to this idea, it was recently shown in mouse models of Lafora disease (progressive myoclonus epilepsy) that defective autophagy accompanied the formation of Lafora bodies, a poorly branched, excessively phosphorylated form of insoluble glycogen [54]–[57]. This finding could have important implications for treatment of Lafora disease as well as for Andersen disease (glycogen branching enzyme deficiency or GSD type IV) and Tarui disease (GSD type VII), which are also associated with the accumulation of polyglucosan aggregates.

The recent proteomic analysis of the autophagy interaction network in human cells by Behrends et al. (2010) [44] identified the muscle form of glycogen synthase as an Atg8-binding protein. This raises the possibility that GlyS could itself act as an adaptor between glycogen and the autophagy machinery. Consistent with this, we found that tagged forms of *D. melanogaster* GlyS and Atg8 colocalized *in vivo* in the muscle during starvation and CQ-induced autophagy (Figure 6). Furthermore, co-immunoprecipitation experiments indicated that the two proteins only form a complex in starved muscles (Figure 6B). As the binding of Gys1 to Atg8 is dependent on the nutritional state of the animal, it is possible that posttranslational modifications such as the inhibiting phosphorylation of GSK3 or the activating binding of Glucose-6-phosphate might regulate this interaction. Consistent with this view, we found that mutation of R593A inhibited the interaction with Atg8 (Figure 6B,D). In yeast the analogous mutation (R581A) generates an enzyme with a lower level of activity than wild type [58],[59]. Along with the fact that the S651A mutation failed to disrupt the GlyS–Atg8 interaction, this suggests that active GlyS is better able to interact with the autophagosome.

Unfortunately, as GlyS is required for the synthesis of glycogen, we could not test its function as a cargo receptor by simple knockdown of the gene. We were, however, able to analyze the ability of the W609A mutant to interact with and localize with Atg8. This mutation disrupts one of the putative LIR motifs conserved between *D. melanogaster* GlyS and its mammalian orthologs. We found that unlike WT GlyS, W609A mutants failed to interact with Atg8 upon starvation, supporting the notion that the DWRTL sequence is a bone fide LIR motif. In yeast glycogen synthase, the residue corresponding to W609 lies in a loop region between two alpha helices, facing inward toward the center of the tetrameric protein [59]. This is a relatively highly ordered region of the protein, such that mutation of W609 could disrupt the function of the protein, inhibiting its activity. We have not ruled out this possibility, but we note that there were no obvious differences in antiglycogen immunostains from WT GlyS or W609A muscles (Figure S4), suggesting that glycogen synthesis was not impaired by overexpression of the mutant.

Nonetheless, we found that GlyS has a general role in promoting autophagy in muscles from CQ-treated animals (Figure 5). The effect of *GlyS* knockdown on autophagosome size could simply be due to the absence of glycogen substrate in these muscles. Alternatively, this finding might indicate that physical interaction between the autophagic machinery and GlyS/glycogen is required for the formation of enlarged autophagosomes, or that the absence of GlyS alters a signaling pathway leading to suppression of autophagosome growth. Recently, it was reported that the AMPK complex, which is known to promote autophagy and phosphorylate GlyS, binds to and is activated by glycogen [60],[61]. A change in AMPK activity and/or localization might therefore play a role in the decreased autophagosome size observed in the *GlyS* knockdown muscle. Interestingly, we observed that much of the Venus–GlyS protein appears to localize to the surface of the Atg8 punctae induced by CQ treatment (Figure 6C,E). It is possible that GlyS residing on the outer surface of the autolysosome could itself sense glucose released via lysosomal glycogen degradation. This would provide a means for feedback from glycogen autophagy to the metabolic and signaling functions of GlyS.

In conclusion, this study represents an advance in our understanding of the role of autophagy in glycogen metabolism in skeletal muscle. The relevance of these processes to animal health, and our investigation of the interaction between GlyS and autophagy, suggests that the *D. melanogaster* model can identify important participants in glycogen autophagy and related myopathies.

## Materials and Methods

### Fly Stocks, Feeding Protocol, and Chloroquine Treatment

Details on fly strains can be found in Text S1. Unless otherwise noted, fly crosses and larvae were maintained in vials containing “standard” cornmeal/soy flour/yeast fly food (see Text S1 for further details). For starvation experiments third instar larvae were individually selected and no more than 20 per experiment were transferred to “low-nutrient food” composed of 0.3× standard food. Likewise, for the rich-food diet, no more than 20 larvae were transferred to “high-nutrient food” composed of standard food supplemented with 100 g/L sucrose and 50 g/L yeast. For each genotype, at least four larvae from at least two independent vials were analyzed. For drug treatments, chloroquine diphosphate salt (Sigma) was added to the food at 2.5 mg/ml.

### Larval Locomotor Assays

Assays were based on previously published methods [62]. For the crawling assay, larvae were positioned at one end of a furrow on the surface of a sylgard plate, with a yeast ball at the far end. The time to crawl 3 cm was measured three times, with the final successful trial used as data for analysis. Trials in which the larva crawled over the edge of the lane were considered unsuccessful, and the larvae were reset at the starting point. For the righting assay, a larva was placed on a sylgard plate, allowed to acclimate for 10 s, then turned upside down. The time it took for the animal to turn back onto its ventral surface was recorded. For both locomotor assays, 10 individual larvae were tested, and *p* values calculated using Student's *t* test.

### Immunostaining, Confocal and Electron Microscopy, and Image Analysis

For whole-mount immunostaining of fly tissues, third instar larval body wall muscles were dissected according to [63] or third instar fat body were dissected and fixed for 15 min in PBS with 4% formaldehyde. After washing in PBT, samples were incubated overnight with appropriate primary and secondary antibodies. Image analysis was done with ImageJ. Transmission electron microscopy was performed using a technique similar to [64]. See Text S1 for further details and list of antibodies used.

### Periodic Acid-Schiff (PAS) Stain

Third instar larvae were fed on standard food, then immersed in 4% formaldehyde in PBS for 1 hr at room temperature, pierced on their posterier end to allow fixative to permeate the tissues, and transferred to 4°C overnight. The tissue was dehydrated in an ethanol series followed by xylene, then embedded in paraplast, and sectioned at 5 µm. Samples were deparrafinized and rehydrated, then stained with PAS, and counterstained with Acidified Harris Hematoxylin following the manufacturer's protocol (Polysciences). Images were collected with an Axiophot 2 compound microscope.

### Glycogen Measurements

Larvae were put on a rich-food diet for 24 h prior to the experiment, then switched to the starvation diet, with or without CQ, for the indicated time. We adapted a protocol used previously for measuring glycogen content in *D. melanogaster* larvae [65]. For each measurement 20 larvae were homogenized on ice in 100 µl PBS, then heat treated at 70°C for 5 min. Homogenate was then diluted 1∶10 in PBS, centrifuged at 12,000 rpm for 3 min, the supernatant was collected, and the glucose analyzed by absorbance at 340 nm using the Glucose (HK) Assay Kit (Sigma). To calculate glycogen levels, untreated samples were compared to samples supplemented with 1 µ/mL amyloglucosidase (Sigma), which degrades the glycogen to glucose. Glycogen levels were then normalized to protein levels in the corresponding homogenate, calculated by Bradford assay, and the ratios for each genotype or treatment were compared using Student's *t* test.

### Generation of UAS-GlyS Transgenes

Glycogen Synthase cDNA LD46952 was cloned into Gateway entry vectors according to the pENTR Directional TOPO Cloning system (Invitrogen), then cloned into destination vectors derived from the *Drosophila* Gateway Collection, and obtained from the *Drosophila* Genomics Resource Center (pTVW = N-terminal Venus tag under the control of the UASt promoter). Point mutations were introduced using the QuikChange II site-directed mutagenesis kit (Stratagene). Atg8a cDNA LD05816 was cloned into pTFW = N-terminal 3xFlag tag under the control of the UASt promoter, as above. Integration into the genome was performed using standard methods. See Text S1 for further details.

### Co-Immunoprecipitations and Western Blot

Prior to dissection larvae were fed in standard food, then eight larvae of each genotype were transferred to new vials containing either standard food or low-nutrient food. Third instar larvae were dissected to obtain a clean carcass with only muscles remaining attached. Dissections were collected in lysis buffer (25 mM Tris–HCl [pH 7.5], 150 mM NaCl, 5 mM EDTA, 1% [v/v] NP-40, 5% [v/v] glycerol, 1× EDTA-free protease and phosphatase inhibitor cocktail [Thermo Scientific]). After homogenization, debris was removed by centrifuging once at 1,200× g for 5 min and once at 1,3000× g for 5 min. Extracts were cleared by incubation with agarose resin (50 µl of packed beads per IP; Thermo Scientific) for 1 h at 4°C, followed by centrifugation at 13,000× g for 15 min. Immunocomplexes were formed by incubation for 2 h at 4°C using anti-GFP nanobodies coupled to agarose beads (10 µl of packed beads per IP; ChromoTek). All washes were in lysis buffer. Western blot was performed using standard protocols. Antibodies used are: mouse anti-GFP (1∶1,000 Abcam ab1218), mouse anti-Tubulin (1∶5,000 Sigma-Aldrich T6199), and rabbit anti-Flag (1∶2,000 Sigma).

## Supporting Information

Figure S1 **Autophagy genes are required for the formation of autophagosomes in CQ-treated larvae.** (A–L) Longitudinal muscles from third instar larvae, expressing *GFP*–*Atg8* under control of the *Dmef2*–*Gal4* driver. (A) *white* RNAi control larvae, starved on low-nutrient food for 6 h+2.5 mg/ml chloroquine (CQ), accumulate large GFP-labeled autophagosomes. (B–C) *Atg1 RNAi* completely blocks autophagosome formation in starved (B) and starved +CQ (C) animals. (D) *Atg5 RNAi*, (E) *Atg12 RNAi*, (F) *Atg2 RNAi*, (G) *Atg9 RNAi*, (H) *Atg16 RNAi*, (I) *Atg3 RNAi*, (J) *Atg6 RNAi*, (K) *Atg18 RNAi*, and (L) *CG1347 RNAi* all block autophagosome formation due to starvation +CQ treatment.
(TIF)

Figure S2 **CQ-induced autophagy does not target mitochondria, filamin, or ubiquitin-labeled aggregates.** (A–C) *Dmef2*–*Gal4*, *UAS*–*GFP*–*Atg8* animals were starved on low-nutrient food for 6 h +2.5 mg/ml CQ, then dissected and assayed for GFP localization. (A) There was no colocalization between GFP–Atg8-labeled vesicles (green) and mitochondria detected by antimitochondrial membrane ATP synthase (red). (B) There was no colocalization between GFP–Atg8-labeled vesicles (green) and the sarcomeric protein filamin (red). (C) The majority of GFP–Atg8-labeled vesicles (green) did not colocalize with ubiquitin (red), although we did occasionally observe some overlap (arrows).
(TIF)

Figure S3 **Quantification of RNAi knockdown.** *GlyS*, *GlyP*, and *Atg1* mRNA expression levels in the third instar larval muscle were analyzed by quantitative RT-PCR (see Text S1). *UAS*–*RNAi* lines were crossed to Dmef2–Gal4. mRNA levels from knockdowns were normalized to *UAS*–*white RNAi* controls. Error bars indicate the SEM.
(TIF)

Figure S4 **Glycogen accumulation in W609A mutant.** *UAS*–*Venus*–*GlyS(WT or W609A mutant)/+;Dmef2*–*Gal4/+* larvae were fed on high-nutrient food, then immunostained with antiglycogen antibody (red) and DAPI (blue). Glycogen accumulates in muscles overexpressing WT GlyS (A) and in muscles overexpressing GlyS (W609A).
(TIF)

Text S1 **Supplemental experimental procedures (fly stocks and feeding protocol, immunostaining and antibodies, image analysis, transmission electron microscopy, generation of transgenic flies, qPCR) and references for supplemental data and experimental procedures.**
(DOC)

## Abbreviations

AMPK: AMP-activated protein kinase
Atg: autophagy related gene
AVM: autophagic vacuolar myopathy
CQ: chloroquine
EM: electron microscopy
FIP200: focal adhesion kinase family interacting protein of 200 kD
G-6-P: glucose-6-phosphate
GAA: acid alpha-glucosidase
GABARAPL1: gamma-aminobutyric acid receptor-associated protein L1
GFP: green fluorescent protein
GlyP: glycogen phosphorylase (D. *melanogaster*)
GlyS: glycogen synthase (D. *melanogaster*)
GSK3B: glycogen synthase kinase 3B
GYS1: muscle glycogen synthase (mammalian)
HOPS: homotypic fusion and vacuole protein sorting
HRP: horse radish peroxidase
LIR: LC3-interacting region
PYGL: liver isoform of mammalian glycogen phosphorylase
PYGM: muscle isoform of mammalian glycogen phosphorylase
Rap: rapamycin
RB1CC1: RB1-inducible coiled-coil 1
Rheb: Ras homolog enriched in brain
RNAi: RNA interference
SNARE: soluble NSF attachment protein receptor
Tor: target of rapamycin
Tsc: tuberous sclerosis complex
Ulk: unc-51-like kinase
Vps34: vacuolar protein sorting-associated protein 34
WT: wild type
