# Supplementary figures and images for: Role of Autophagy in Glycogen Breakdown and Its Relevance to Chloroquine Myopathy
Source: PLoS Biol. 2013 Nov 12;11(11):e1001708. doi: 10.1371/journal.pbio.1001708 (PMC3825659; doi:10.1371/journal.pbio.1001708)

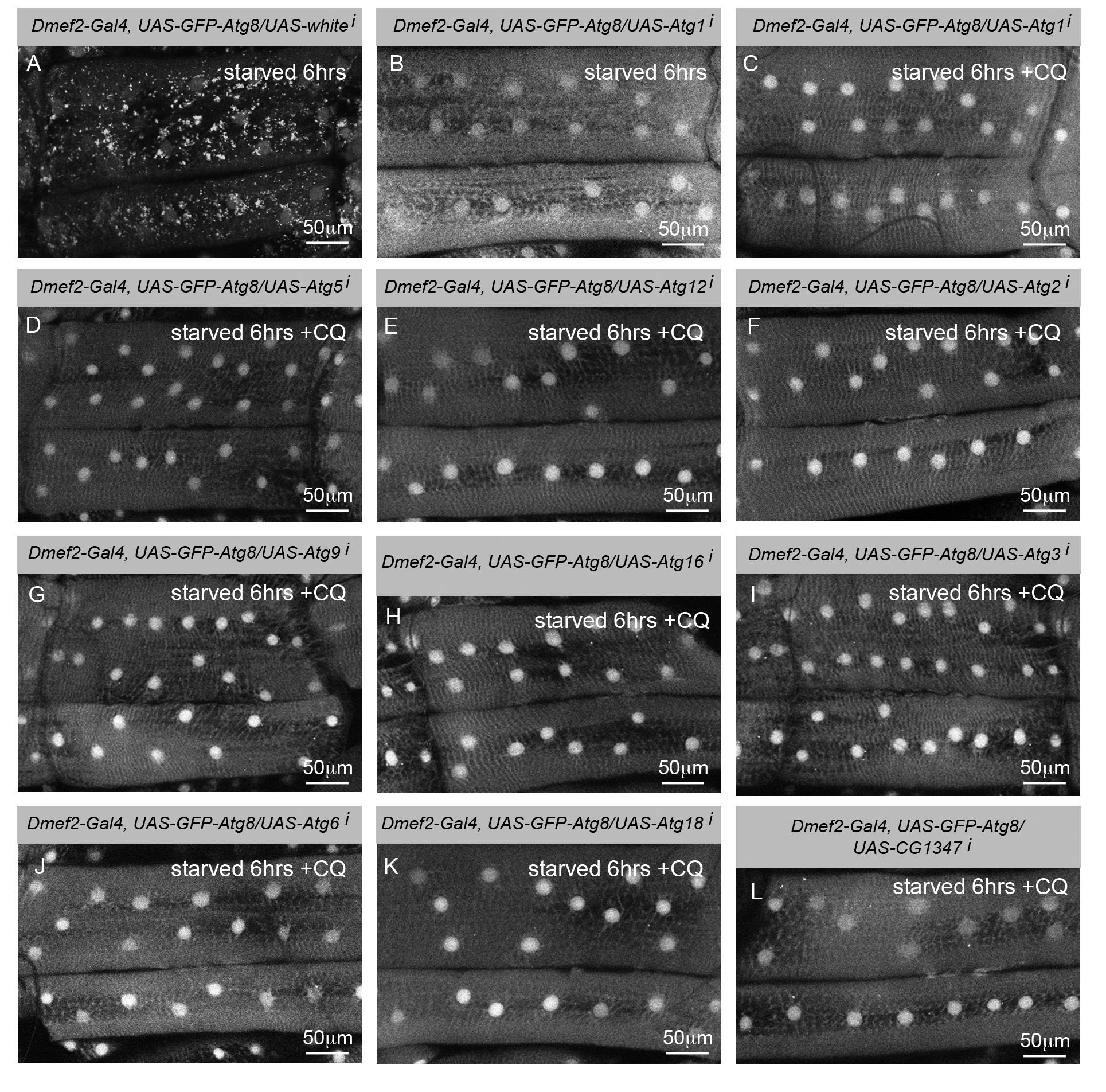

Supplement: Figure S1 — Autophagy genes are required for the formation of autophagosomes in CQ-treated larvae. (A–L) Longitudinal muscles from third instar larvae, expressing GFP–Atg8 under control of the Dmef2–Gal4 driver. (A) white RNAi control larvae, starved on low-nutrient food for 6 h+2.5 mg/ml chloroquine (CQ), accumulate large GFP-labeled autophagosomes. (B–C) Atg1 RNAi completely blocks autophagosome formation in starved (B) and starved +CQ (C) animals. (D) Atg5 RNAi, (E) Atg12 RNAi, (F) Atg2 RNAi, (G) Atg9 RNAi, (H) Atg16 RNAi, (I) Atg3 RNAi, (J) Atg6 RNAi, (K) Atg18 RNAi, and (L) CG1347 RNAi all block autophagosome formation due to starvation +CQ treatment. (TIF) [file pbio.1001708.s001.tif]

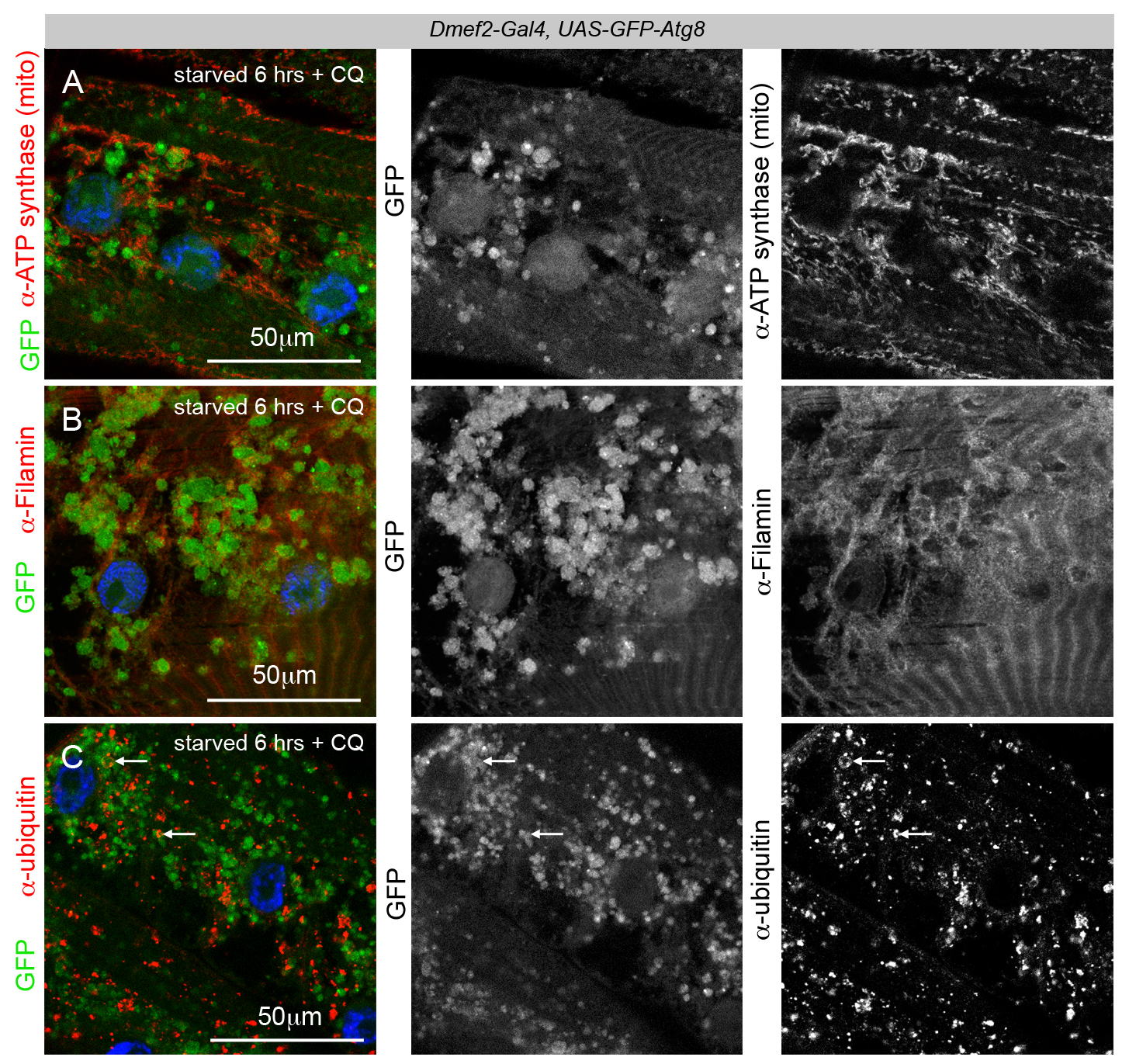

Supplement: Figure S2 — CQ-induced autophagy does not target mitochondria, filamin, or ubiquitin-labeled aggregates. (A–C) Dmef2–Gal4, UAS–GFP–Atg8 animals were starved on low-nutrient food for 6 h +2.5 mg/ml CQ, then dissected and assayed for GFP localization. (A) There was no colocalization between GFP–Atg8-labeled vesicles (green) and mitochondria detected by antimitochondrial membrane ATP synthase (red). (B) There was no colocalization between GFP–Atg8-labeled vesicles (green) and the sarcomeric protein filamin (red). (C) The majority of GFP–Atg8-labeled vesicles (green) did not colocalize with ubiquitin (red), although we did occasionally observe some overlap (arrows). (TIF) [file pbio.1001708.s002.tif]

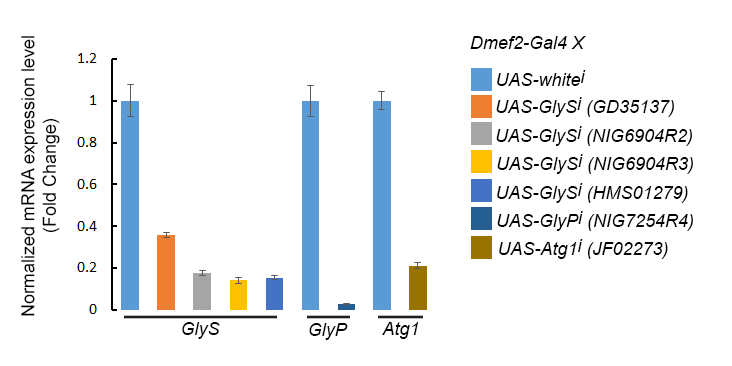

Supplement: Figure S3 — Quantification of RNAi knockdown. GlyS, GlyP, and Atg1 mRNA expression levels in the third instar larval muscle were analyzed by quantitative RT-PCR (see Text S1). UAS–RNAi lines were crossed to Dmef2–Gal4. mRNA levels from knockdowns were normalized to UAS–white RNAi controls. Error bars indicate the SEM. (TIF) [file pbio.1001708.s003.tif]

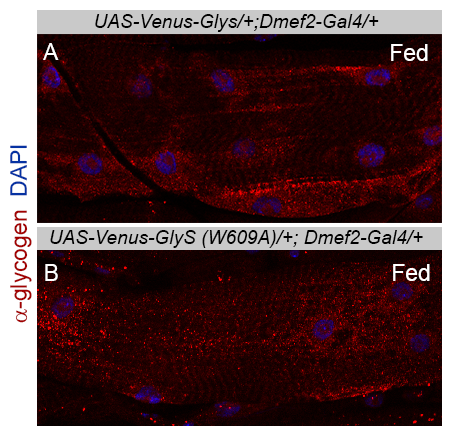

Supplement: Figure S4 — Glycogen accumulation in W609A mutant. UAS–Venus–GlyS(WT or W609A mutant)/+;Dmef2–Gal4/+ larvae were fed on high-nutrient food, then immunostained with antiglycogen antibody (red) and DAPI (blue). Glycogen accumulates in muscles overexpressing WT GlyS (A) and in muscles overexpressing GlyS (W609A). (TIF) [file pbio.1001708.s004.tif]
