# Supplementary material for: Role of Autophagy in Glycogen Breakdown and Its Relevance to Chloroquine Myopathy
Source: PLoS Biol. 2013 Nov 12;11(11):e1001708. doi: 10.1371/journal.pbio.1001708 (PMC3825659; doi:10.1371/journal.pbio.1001708)
Supplement: Text S1 — Supplemental experimental procedures (fly stocks and feeding protocol, immunostaining and antibodies, image analysis, transmission electron microscopy, generation of transgenic flies, qPCR) and references for supplemental data and experimental procedures. (DOC) [file pbio.1001708.s005.doc]

**Supplemental Information**

**Supplemental Experimental Procedures**

**Fly Stocks and Feeding Protocol**

Fly stocks used were: *yw; Dmef2-Gal4* *; UAS-GFP-Atg8a*  was recombined with *Dmef2-Gal4; UAS-HRP-Lamp1* (gift of H. Kramer, University of Texas Southwestern Medical Center, Dallas, TX); *UAS-Rheb* (Bloomington #9688) ; *CG6904[MI01490]* GFP gene trap line (Bloomington # 34440). The following RNAi lines were obtained from the DRSC/TriP DRSC/TRiP at Harvard Medical School (http://www.flyrnai.org/TRiP-HOME.html): *Glycogen synthase* (HMS01279); *Tsc1* (JF01484); *gigas* (HM04083); *white* (HMS0004);  *Atg1* (JF02273);  *Atg2* (JF02786); *Atg3* (HMS01348); *Atg5* (HMS01244); *Atg6* (HMS01483); *Atg9* (JF02891); *Atg12* (HMS01153); *Atg16/CG31033* (HMS01347); *Atg18* (JF02898), and *CG1347* (HMS01611). Additional RNAi lines were obtained from the NIG Japan: *GlyS* (6904R-2), *GlyS* (6904R-3), and *GlyP* (7254R-4); and *GlyS* (GD35137) from the VDRC. Unless otherwise noted, flies were reared at 25°C and fed with ‘standard’ food composed of 16.5 g/L yeast, 9.5 g/L soy fluor, 71 g/L cornmeal, 5.5 g/L agar, 5.5 g/L malt, 7.5% corn syrup, 0.4% propionic acid, and 1.3% Tegosept.

### Immunostaining and Antibodies

After fixation in 4% formaldehyde, dissected tissues were washed in PBT and incubated overnight using the following antibodies: mouse anti-Poly-Ubiquitin, 1:300 (FK2; Enzo life sciences); mouse anti-glycogen, 1:100 ; mouse anti-ATP synthase subunit alpha, 1:300 (MS507, Mitosciences); rat anti-filamin, 1:1000 . After incubation with primary antibodies, the samples were washed in PBT and incubated with Alexa-conjugated secondary antibodies (Molecular Probes, 1:1000) and/or Alexa 635-conjugated phalloidin (1:1000) to visualize F-actin. Nuclei were visualized by DAPI staining (1μg/ml). Samples were washed in PBT and mounted in 1:1 glycerol/PBS and images were acquired with a Leica SP2 laser scanning confocal microscope.

**Image analysis**

For quantification of autophagy area, z-stacks at 40X of single larval longitudinal muscles were obtained by confocal microscopy. ImageJ was used to produce a maximum intensity projection of the stack. Thresholds on the GFP-Atg8 channel allowed for the elimination of background signal not beloning to any autophagosomal structure, and the area of the remaining signal was calculated to determine the autophagic area per muscle. Five ventral longitudinal muscles from individual animals were analyzed for each genotype. For quantitation of autophagic vesicle size and number single confocal sections were obtained at 63X at the level of the nucleus. ImageJ was used to count and measure the mean size of GFP-Atg8 and HRP-LAMP puncta in the region surrounding a single nucleus from the ventral longitudinal muscles of ten individual animals. For all image analysis p-values were calculated by Student’s t-test.

**Transmission Electron Microscopy**

For electron microscopy, *Dmef2-Gal4/UAS-whitei* or *Dmef2-Gal4, UAS-GFP-Atg8* 3^rd^ instar larvae were first starved on low nutrient food for 6 hr + or – 2.5 mg/ml CQ. Larvae were then dissected as for immunofluorescence, but fixed using 2% formaldehyde/2,5% gluteraldehyde in 0.1 sodium cacodylate buffer (pH 7.4) for 2 hr. After washes with cacodylate buffer, samples were treated with 1% Osmiumtetroxide/1.5% Potassiumferrocyanide (in water) for 1 hr at room temperature. After washes with water samples were treated with 1% Uranyl acetate (in water) for 30 min. Dehydration was done with consecutive washes with 70%, 90% and 100% ethanol.

Propyleneoxide infiltration was done for 1 hr and infiltration with Epon was performed o/n at 4⁰C. From here, samples were sectioned using a Reichert Ultracut. Sections were contrasted with uranyl acetate and lead citrate prior to image collection on a JEOL 1200EX 80kV electron microscope (Harvard Medical School EM Facility).

**Generation of transgenic flies**

For Gateway cloning, *GlyS* was amplified from cDNA clone LD46952 using the following PCR Primers: (Forward) 5’-CACCATGAATCGTCGCTTTTCG-3’; (Reverse, C-tagged constructs) 5’-CTTAATCCCGAATTCCTTGAG-3’; (Reverse, N-tagged constructs) 5’-CTACCCAATCCCCAATTCC-3’.

**qPCR:**

For each genotype muscles from 10 3^rd^ instar animals were dissected off of the cuticle and RNA was prepared with Trizol (Invitrogen), followed by purification with the RNeasy kit (Qiagen). Complementary DNA (cDNA) was synthesized with the iScript cDNA Synthesis kit (Bio-Rad), and quantitative RT-PCR was performed with the iQ SYBR Green Supermix (Bio-Rad). Rp49 was used as normalization reference. Relative quantitation of mRNA expression was calculated using the comparative *C*_T_ method. The primers used were as follows: rp49, 5'-ATCGGTTACGGATCGAACAA-3' (forward) and 5'-GACAATCTCCTTGCGCTTCT-3' (reverse); GlyS, 5’-TCGCTTTTCGAGAGTGGAGTC-3’ (forward) and 5’ ATATGCCTTCGACCGGATCAC-3’ (reverse); GlyP, 5’-ATGGCTATGGCATCCGTTATG-3’ (forward) and 5’-CATGGCAAACACCCTTTGGG-3’ (reverse); Atg1, 5’-CTAAAGCCGTCGTCCAATGT-3’ (forward) and 5’-GAACAGCATGCTCCGGTATT-3’ (reverse).

**Supplemental References**

1. Ranganayakulu G, Schulz RA, Olson EN (1996) Wingless signaling induces nautilus expression in the ventral mesoderm of the Drosophila embryo. Dev Biol 176: 143-148.

2. Juhasz G, Hill JH, Yan Y, Sass M, Baehrecke EH, et al. (2008) The class III PI(3)K Vps34 promotes autophagy and endocytosis but not TOR signaling in Drosophila. J Cell Biol 181: 655-666.

3. Patel PH, Thapar N, Guo L, Martinez M, Maris J, et al. (2003) Drosophila Rheb GTPase is required for cell cycle progression and cell growth. J Cell Sci 116: 3601-3610.

4. Venken KJ, Schulze KL, Haelterman NA, Pan H, He Y, et al. (2011) MiMIC: a highly versatile transposon insertion resource for engineering Drosophila melanogaster genes. Nat Methods 8: 737-743.

5. Baba O (1993) [Production of monoclonal antibody that recognizes glycogen and its application for immunohistochemistry]. Kokubyo Gakkai Zasshi 60: 264-287.

6. Sokol NS, Cooley L (2003) Drosophila filamin is required for follicle cell motility during oogenesis. Dev Biol 260: 260-272.

7. Zheng L, Michelson Y, Freger V, Avraham Z, Venken KJ, et al. (2011) Drosophila Ten-m and filamin affect motor neuron growth cone guidance. PLoS One 6: e22956.
